# Prevalence and clinical features of *Cryptosporidium* infection in hemodialysis patients 

**Published:** 2017

**Authors:** Mohammad Ali Mohaghegh, Seyed Hossein Hejazi, Mohsen Ghomashlooyan, Hamed Kalani, Farzaneh Mirzaei, Mehdi Azami

**Affiliations:** 1 *Department of Laboratory Sciences, Torbat Heydariyeh University of Medical Sciences, Torbat Heydariyeh, Iran*; 2 *Skin Diseases and Leishmaniasis Research Center, Isfahan University of Medical sciences, Isfahan, Iran*; 3 *School of Paramedicine, Shahid-Sadoughi University of Medical Science, Yazd, Iran *

**Keywords:** Cryptosporidium, Hemodialysis, Diarrhea, Immunocompromised patients, Prevalence

## Abstract

**Aim:**

This cross-sectional study aims to assess the prevalence of *Cryptosporidium* in hemodialysis patients compared with healthy individuals in central Iran from August 2014 to January 2015.

**Background:**

Cryptosporidiosis is a major cause of acute and persistent diarrhea with significant morbidity and mortality in immunocompromised patients such as those undergoing renal dialysis.

**Methods:**

Three stool samples were collected from 330 hemodialysis patients and 150 healthy individuals on 3 consecutive days. The samples were screened for *Cryptosporidium* infection using formalin-ether sedimentation and modified Ziehl-Neelsen staining. Demographic variables as well as risk factors were recorded.

**Results:**

Out of 330 dialysis patients and 150 healthy individuals, 10 (3%) and 1 (0.7%) were infected with *Cryptosporidium*, respectively. We found statistically significant differences between infection and place of residency, hygiene status, education level, diarrhea, and abdominal pain in the two groups (p<0.05). On the other hand, there was no relationship between infection and sex, contact with domestic animals, fever, vomiting, nausea, flatulence, anorexia, duration of dialysis and underlying disorders in the two groups. Also, there was a statistically significant difference between age and infection in hemodialysis patients (p=0.003). A higher infection rate was observed in patients under 20 years of age.

**Conclusion:**

Risk factors for *Cryptosporidium* infection must be controlled. We strongly recommended that stool samples from such patients, especially those with severe or prolonged diarrhea, should be examined with modified Ziehl-Neelsen staining for appropriate and timely treatment.

## Introduction

*Cryptosporidium* is a coccidian protozoan parasite with gastrointestinal manifestations in humans and animals; it is predominantly transmitted via the fecal-oral route (1). Numerous *Cryptosporidium* species, including *C. hominis, C. parvum, C. meleagridis, C. canis, C. wrairi, C. suis, C. felis, C. andersoni and C. muris*, can infect humans (2-5). Cryptosporidiosis is spread worldwide and its prevalence is high in developing countries (6). *Cryptosporidium* causes watery or mucoid diarrhea with abdominal pain that may persist from a few days to much longer (7). Although benign and self-limiting in immunocompetent individuals, cryptosporidiosis is a major cause of acute and persistent diarrhea with significant morbidity and mortality in immunocompromised patients such as those with HIV/AIDS, malignancies, organ transplantation and those undergoing renal dialysis (1, 8, 9). 

Chronic renal failure (CRF) is one of the causes of insufficient immune response to infections. In patients undergoing hemodialysis, sepsis-related death is 100 to 300-times more frequent than healthy individuals (10). Progressive and irreversible loss of renal functions causes uremia (11), which impairs T-cell activation and compromises antibody production (12). Malnutrition and vitamin deficiency occur due to either inadequate intake or loss during dialysis. Also during dialysis, prealbumin is decreased and C-reactive protein is increased in patients (13-15). Repeated per-dialysis hypotension, with the activation of nitric oxide, platelet dysfunction and anemia result in specific immunological changes (16, 17). Leukocyte dysfunction, increased susceptibility to opportunistic infection such as *Cryptospodidium* spp., and impaired neutrophil activity (phagocytosis, migration, bactericidal) also occur in hemodialysis patients (18, 19). 

The present study aims to assess the prevalence of *Cryptosporidium* spp. in a group of hemodialysis patients and compared with healthy individuals in central Iran. 

## Methods

This cross-sectional study was conducted from August 2014 to January 2015 at four hospitals (Shariati, Nor, Al-Zahra, Hojjatieh) in Isfahan, central Iran. Stool specimens were collected from 330 hemodialysis patients and 150 healthy individuals (i.e. individual’s working in many wards of the above hospitals) as the control group. Informed consent was obtained from all patients for participation in the study and the study was conducted in accordance with the principles of the Declaration of Helsinki. Demographic variables, as well as risk factors, were recorded, including sex, age, place of residence, close contact with domestic animals, hygiene status, education, fever, gastrointestinal symptoms, duration of dialysis and underlying disorders. 

Three stool samples were collected from each participant in sterile plastic containers on 3 consecutive days. The samples were preserved in 10% formalin and kept in a cool and dry place until examination. The stool samples were concentrated using formalin-ether method. From each sample, a separate thin microscopic smear was prepared and allowed to dry in air. The smears were fixed with absolute methanol and then stained by cold modified Ziehl-Neelsen staining. Finally, the air-dried slides were examined with light microscopy for presence of *Cryptosporidium* spp. oocysts at 1000× magnification. The data were analyzed with chi-square test, and P-values less than 0.05 were considered statistically significant. 

## Results

Out of 330 dialysis patients and 150 healthy individuals, 10 (3%) and 1 (0.7%) were infected with Cryptosporidium spp., respectively. The mean age was 33.1 ± 12 years in the infected patients group and 34 ± 11 years in the infected healthy group. We found a statistically significant difference in age among the hemodialysis patients; a higher infection rate was observed in patients under 20 years of age. There was a significant correlation between the prevalence rate of *Cryptosporidium* infection and place of residence in the patient and control groups (p<0.05). *Cryptosporidium* infection rate in the rural population (13.7%) was higher than the urban population (2.3%) in the hemodialysis patients group. A similar condition was seen in healthy individuals group. Five (4%) of hemodialysis patients and 1 (1.8%) of healthy individuals who were infected with Cryptosporidium had diarrhea which indicates a significant difference. Also, there was a statistically significant difference between abdominal pain and Cryptosporidium infection (p=0.028). 

**Table 1:** Demographic characteristics of hemodialysis patients and healthy individuals infected with *Cryptosporidium* spp

	Case group (n=330)			Control group (n=150)		
	Total	Infected n (%)	*P* value	Total	Infected n (%)	*P* value
Sex:						
Male Female	207 123	6 (2.9) 4 (3.3)	0.243	90 60	1 (1.2) 0 (0)	0.186
Age (year)						
≤ 20 21-65 ≥ 65	130 100 100	5 (3.9) 2 (2) 3 (3)	0.003	50 50 50	1 (2) 0 (0) 0 (0)	0.061
Place of residency						
Urban Rural	308 22	7 (2.3) 3 (13.7)	0.001	138 12	1 (0.7) 0 (0)	0.003
Contact with domestic animals						
Yes No	148 182	4 (2.8) 6 (3.3)	0.261	49 101	0 (0) 1 (0.9)	0.183
Hygiene status						
Excellent Moderate Bad	210 90 30	3 (1.4) 3 (4.5) 3 (10)	0.003	60 60 30	0 (0) 0 (0) 1 (3.4)	0.034
Education						
Illiterate Elementary education High school diploma or higher	55 95 180	5 (9.1) 3 (3.2) 2 (1.2)	0.002	18 34 98	1 (5.6) 0 (0) 0 (0)	0.034

**Table 2:** Clinical features of hemodialysis patients and healthy individuals infected with *Cryptosporidium* spp

	Case group (n=330)			Control group (n=150)		
	Total	Infected n (%)	P value	Total	Infected n (%)	P value
Fever						
Yes No	105 225	3 (2.9) 7 (3.2)	0.82	54 96	0 (0) 1 (1.1)	0.15
Diarrhea						
Yes No	125 205	5 (4) 5 (2.5)	0.03	93 57	1 (1.1) 0 (0)	0.01
Vomiting						
Yes No	168 162	6 (2.4) 4 (2.5)	0.35	5 145	0 (0) 1 (0.7)	0.17
Nausea						
Yes No	123 207	3 (2.5) 7 (3.4)	0.56	41 109	0 (0) 1 (1.3)	0.15
Flatulence						
Yes No	281 49	6 (2.2) (8.2)	0.26	68 82	0 (0) 1 (1.5)	0.83
Anorexia						
Yes No	245 85	6 (2.5) 4 (4.8)	0.38	62 88	0 (0) 1 (1.2)	0.35
Abdominal pain						
Yes No	205 107	7 (3.5) 3 (2.9)	0.03	48 102	1 (2.1) 0 (0)	0.03

The prevalence rate of *Cryptosporidium* infection did not correlate with sex, contact with domestic animals, fever, vomiting, nausea, flatulence, anorexia, duration of dialysis and underlying disorders in either group (p>0.05). The main demographic and clinical parameters of the study groups are shown in tables 1 and 2. The underlying disorders and duration of dialysis in hemodialysis patients are shown in Table 3. 

## Discussion

Studies have shown that damage to the immune system in patients with CRF often leads to increased susceptibility to bacterial infections, mainly involving the respiratory, digestive, urinary systems and skin. These infections are responsible for between 14-40% of deaths in these patients (10, 20). 

In addition, studies have reported high infection rate with Cryptosporidium spp. in hemodialysis patients (21-23). 

**Table 3:** Disorder and duration of dialysis recorded in hemodialysis patients infected with *Cryptosporidium* spp

	Case group (n=330)			
	Total	Infected n (%)	*P* value
Duration of dialysis (month)			
1-12 13-24 ≥ 25	101 104 125	4 (3.9) 3 (2.9) 3 (2.4)	0.84
Underlying disorders			
Diabetes Hypertension Polycystic kidney Glomerulonephritis Renal stone	188 204 89 56 61	6 (3.2) 9 (4.5) 4 (4.5) 2 (3.6) 1 (1.7)	0.68

In the present study, the rate of infection with Cryptosporidium was 3%. Previous studies have reported the prevalence of cryptosporidiosis in hemodialysis patients from different regions of the world to be 20.7%, 15%, 26.4% and 11.5% in Turkey, Egypt, Brazil and Iran, respectively (21, 22, 24, 25). There was a statistically significant difference between age and *Cryptosporidium* infection in the patients group (p=0.003) and a higher infection rate was observed in patients under 20 years of age, while there was no relationship between age and *Cryptosporidium* infection in the healthy individuals (p=0.061). We found a statistically significant difference in age among the hemodialysis patients. Our result is similar to a previous study (26) and is inconsistent with the findings of Raja et al. (2014) and Seyrafian et al. (2006). 

Malnutrition is a sign in hemodialysis patients (13) that causes geophagia, especially in children, which raises the risk of infection with *Cryptosporidium* spp. (28). In the present study, there was a significant correlation between the prevalence rate of Cryptosporidium infection and place of residence in the patient and control groups (p<0.05). The results of recent studies are similar to our finding (26). Socioeconomic status and close contact with domestic animals, especially sheep and cattle, in rural residents increase the risk of infection with intestinal parasites (29). Sheep and cattle play an important role in the spread of infection and an increased risk of infection with *Cryptosporidium parvum* in humans (30). 

In our study, the poverty and illiteracy status were considered as two risk factors in relation to Cryptosporidium infection in hemodialysis patients that are similar to the result of a recent study (26). Contrary to this result was a study from Pakistan which examined 644 fecal specimens from renal transplant patients with acute diarrhea and found no significant difference between *Cryptosporidium* infection and age, gender or duration of dialysis (27). The insufficient information regarding risk factors in illiterate patients, such as consumption of unsafe water and food, is associated with an increased risk of the acquisition of cryptosporidial infection (31). Gastrointestinal symptoms, especially diarrhea (chronic or acute), nausea, hyporexia, vomiting, and abdominal pain are very common in patients undergoing dialysis (32). A high prevalence of diarrhea was observed in the hemodialysis patients with *Cryptosporidium* in our study. Cryptosporidiosis is a major cause of acute and persistent diarrhea, especially in immunocompromised individuals, malnourished children and the elderly in developing countries (6). Seyrafian et al. (2006) compared the prevalence rate of *Cryptosporidium* infection in hemodialysis patients and 2 control groups (i.e., their healthy family members and normal population). Stool specimens of 104 adult outpatient chronic hemodialysis patients, their 91 healthy family members, and 140 healthy individuals were examined for the presence of Cryptosporidium oocysts using a modified acid-fast staining method. Twelve (11.5%) dialysis patients were infected with *Cryptosporidium*. This was significantly higher than 4 (4.4%), and 5 (3.6%) cases in the 2 control groups, respectively (p < 0.05). There was no significant difference between the 2 control groups. The prevalence rate of *Cryptosporidium* infection did not correlate with patients' sex, age, duration of dialysis, history of kidney transplantation, or history of taking immunosuppressive drugs. However, it was significantly higher in diabetics vs. nondiabetics (19.4% vs. 8.3%, respectively, p < 0.05). The results indicate that the prevalence rate of *Cryptosporidium* infection is considerably higher in dialysis patients than the general population. Moreover, dialyzed diabetic patients had the highest rate of infection. These finding are in contrast with our results. In this study, diabetes mellitus and hypertension were detected in 57% and 61.8% patients of the hemodialysis group, respectively. Diabetes mellitus is the second major cause of CRF that leads to simultaneous impairment of other organs and the immune system. There is a high prevalence of hypertension in CRF patients, depending on the type of nephropathy, degree of renal failure, advanced age and the presence of diabetes (33). Strict control of blood pressure, hyperglycemia and glycosuria are essential for avoiding and delaying the decrease in renal function (33, 34). 

Finally, according to the findings, *Cryptosporidium* is an opportunistic protozoan and a causative agent of prolonged diarrhea in immunocompromised patients, such as those undergoing hemodialysis. Therefore, it is necessary to control risk factors and adopt preventive measures against infection. We strongly recommended that the stool samples of these patients, especially those with severe or prolonged diarrhea, should be examined with cold modified Ziehl-Neelsen staining for appropriate and timely treatment.
